# Successful ability to stay at home - an interview study exploring multiple diagnosed older persons and their relatives’ experiences

**DOI:** 10.1186/s12877-024-05439-7

**Published:** 2024-10-24

**Authors:** Lena-Karin Gustafsson, Anna Bondesson, Tina Pettersson, Gunnel Östlund

**Affiliations:** 1 Division of Caring Science, School of Health, Care and Social Welfare, Mälardalen University, Box 325, Eskilstuna, RN 63105 Sweden; 2 Division of Social work, School of Health, Care and Social Welfare, Mälardalen University, Eskilstuna, Sweden

**Keywords:** Emotional support, Homecare, Instrumental support, Love, Multi morbidity, Next-of-kin, Older persons, Patient-centred care, Secure care

## Abstract

**Background:**

Society places increased demands on regions and municipalities to jointly carry out activities for multi-diagnosed older persons with extensive coordination needs. Interprofessional collaboration is reported as an important success factor for the overall health care of this group of patients. This project focuses on older persons with multiple diagnoses and their relatives’ own experiences of what is most important for safety and security in their homes. The **aim** of the study was: to illuminate the meaning of success for the ability to stay at home as experienced by older persons with multiple diagnoses and their relatives.

**Methods:**

The project had a descriptive explorative design with a phenomenological hermeneutic approach based on analysis of 14 in-depth interviews with older people and their relatives.

**Findings:**

Own resources were identified such as belief in the future, spiritual belief, social network, having loved ones and pets. Technical aids were seen as helpful resources, working as indoor and outdoor security safeguards. These resources included having good telephone contact with social and professional networks as well as other forms of personal equipment such as a personal alarm. The professional network was a resource, acting as support when the patient’s own abilities were not sufficient. Finally, having personnel who had the time and interest to listen was seen as crucial to experience safety.

**Conclusions:**

The main reason for being able to continue homecare was the person’s self-care system, their personal, social, and technical resources. Professional care development should anchor team work to the patient’s own system of self and informal care.

## Introduction

Society places increased demands on the region and municipality to jointly perform activities for multi-diagnosed older persons with extensive coordination needs. Shorter and fewer hospital stays result in reduced complication risks such as infections and pressure ulcers. Interprofessional collaboration is an important success factor for patients to feel satisfied with care. This research project follows the work of an integrated interprofessional team, as they design new ways of working, offering a coherent health and care system using different care providers for older people with coordination needs in ordinary housing. By creating interprofessional teams, with professions from both region and municipality working together in the older person’s home, the care and concern the person receives can be improved.

## Background

There is growing interest to empower older persons to age in the place of their own choice, most often their own homes [1, 2]. Research shows that older persons prefer to remain in their home as their health care needs intensify [3]. Recent research has extended life expectancy and led to a rise in the number of years living with multimorbidity as well as increased support needs [4]. For example, multimorbidity is shown in 86% of patients diagnosed with heart failure. These other conditions, such as diabetes, COPD, osteoarthritis, cancer, and dementia, affect the individual’s, quality of life and symptom burden which affects home care needs [5]. In Sweden, municipalities are responsible for care in the home, while regions are responsible for health care and treatment. Identifying, illuminating and promoting the success factors for older persons with multimorbidity, enabling safe home care instead of hospital care, remains a persistent challenge. Werner et al. [6] illustrate that future sustainable interventions must always be developed to support older persons and informal caregivers’ resources in managing the demands caused by patients remaining at home. Multi professional teams when several professions, sometimes from different organisations, work together around the same patient constitute a good prerequisite for holistic care, where professionals with different skills have opportunities to complement each other [7, 8]. Home health care is gradually developing to include increasingly advanced health care, to meet increasingly complex needs. Solving more complex needs and problems, means a variety of knowledge is often required by professionals with different competencies working together. As more tasks are moved from hospital to home care, health care technology development can also be included in this complexity [9, 10]. Interprofessional collaboration is needed to ensure the appropriate use of medical devices and other new health care technology often necessary in the care of persons with multiple diagnoses. Health care is most often provided in settings that focus on a single disease while the focus of older persons should be on provision of care and comprehensive care for multiple diseases, even though this may be more difficult [11].

Internationally, there are various health care programmes that provide multi-morbidity care. These programmes vary greatly according to target group, care providers, implementation practices and acute care delivery organisation [12]. Many researchers agree that future care planning should focus on a deeper understanding of the complex challenges patients and their relatives face to attain safety at home [13]. Older persons with multiple diagnoses are also recognized as a particularly vulnerable group, especially dependent on their relatives. Kneck et al. [14] describe the heavy burden on the patient and relatives to keep track of and communicate information between different caregivers when the patient is cared for at home. Their study showed that patients were expected to be active partners in their own home care but were in many cases largely excluded from the information flow concerning their care.

Kirst et al. [15], emphasize the importance of having multi-disciplinary teams in home- and community-based services (HCBS) offering processes of effective communication and knowledge sharing. Home care teams aim to offer support allowing older persons to age in their homes even though they are likely to require long-term care. Some American studies [2] use the HCBS model to predict and explain when community-dwelling older persons’ utilization of HCBS allowed them to remain at home or in community care. Their study identifies two significant supportive factors for older persons to remain at home. These were the use of paid instrumental activities of daily living by personal care services and awareness of unmet needs. Early awareness of unmet needs could lead to a better adaptation contributing to the increased likelihood of older persons’ continuing to remain in their homes.

The overall project has been created in a theoretical perspective of person-centred care, which has come to be seen as one of the core competencies in nursing, as well as central in other professional efforts given to older people in the home. As populations grow older, health care must change focus, from a disease-oriented to a more person-oriented/centred care [12, 16]. The basis for person-centred care consists of values ​​such as respect for the person, the person’s right to self-determination and to create a common understanding and horizon [17]. The person’s mental, physical, and social health and the older person’s own values ​​are integrated in this perspective [18, 19]. Since older multi-ill persons often have a variety of needs a key element in professional care is to work with person-centred care [20]. In addition to a theoretical perspective, person-centred care can also be seen as a way of working to establish health promoting and supportive relationships between caregivers, patients, and other important people in the life of the older multi-diagnosed person.

## Aim

The aim of this specific study was to illuminate the meaning of success for the ability to stay at home according to older persons with multiple diagnoses and their relatives.

## Materials and methods

### Study design

A multidisciplinary research group at the university started a research project in collaboration with a medium-sized municipality in Sweden and their affiliated region in 2020. The project was designed as an intervention and intended to follow a newly created interprofessional team of approximately 18 professionals mixed from both regional and municipal caregiver organisations, with a focus on older persons with multiple diagnoses in great need of health care. The intention was that this interprofessional team would be co-creative and develop new work models to reduce care efforts, promote self-care and a safe situation at home for older persons with multiple diagnoses.

### Participants

The project has a descriptive explorative design with a phenomenological hermeneutic.

approach based on analysis of 14 in-depth interviews from older persons and their relatives. The inclusion criteria were persons with multiple diagnoses with extensive coordination needs within one middle sized city in southern Sweden. During the study the interprofessional team provided care to ten older persons with multiple diagnoses. All matched the inclusion criteria and were invited to the study, as were their close relatives. They were informed about the project orally and in an information letter as they were invited to participate. Ten older persons (*n* = 10, y = 79–95) and four relatives (*n* = 4, y = 51–81) answered the invitation and were contacted by the research team for more information and to organise an interview.

### Data collection

Informed consent was obtained in writing and verbally in connection to participation in the research project from all the older persons and their relatives included in the research project. The participants were informed that participation was voluntary, and that any data collected would be handled to exclude access by unauthorized people. Interviews took place in the participant’s or the relative’s own home and were then recorded digitally and transcribed verbatim. The questions were open and invited the older persons to talk about the main.

topic, such as: *Could you please tell us about what you have experienced as desirable to create a safe*,* secure*,* and sustainable situation in the home? Can you describe the meaning of success for your ability to stay at home instead of being hospitalized for your multi-morbidity i.e. `that a person has two or more diseases at the same time´?* A question for the relatives could be: *Can you identify factors that help your relative to be safe enough to stay at home instead of hospital?* The interviews lasted for ~ 1 h. The study ethics was approved by the Regional Ethics Committee in Uppsala, Sweden (D. nr, 2019–05127).

### Analysis

The interviews were transcribed, analysed, interpreted, and themed according to Lindseth and Norberg’s [21] description of the phenomenological hermeneutic method based on Ricoeur’s [22–24] philosophy of interpretation and understanding. The chosen method for analysis aims at illuminating the meaning of a phenomena i.e. to understand the movement from what the text says, to what it talks about [21]. Analysis started with (a) the construction of a naïve interpretation to obtain an initial understanding of the meaning of the text after a first naïve reading. This is supposed to halt the interpretive process and allow the reader to see the text as new and without theoretical interference. This first naïve interpretation was then validated in the second step by structural analysis. (b) The text was divided into meaning units to convey the essence of the phenomena of interest. These units were then condensed and divided into subthemes and then main themes with names which represented a broader understanding of the meaning of the text. This structural analysis functions as a decontextualization, from the researcher’s subjective world to a common world of experiences as described by Ricoeur [22, 24]. After that a comprehensive interpretation (c) was performed by reading the text, including naive comprehension and structural analysis, as well as reading selected literature. In this case literature comprised earlier research, theoretical frameworks and new literature about things illuminated in the analysis. This helped to achieve what Ricoeur calls re-contextualization of the text to bring new light on the phenomena.

Throughout the structural analysis, the validity of the findings was supported by the researchers in the project group representing four different professional backgrounds (social worker, district/homecare nurse, psychiatric nurse, physiotherapist), different scientific fields (physiotherapy, social work, psychiatric care and primary-home health care) and three academic levels (associate professors, doctor degrees and masters), all with health care experience of older persons with multiple diagnoses living at home.

### Findings

#### Final Naïve interpretation

Success factors for the ability to stay at home were experienced by older persons with multiple diagnoses and their relatives. These included: Own resources like a striving will, hope and spiritual beliefs as well as social resources such as social network support and having close relatives around who could also help, so that the spouse received necessary help. The older persons also pointed out that the company of pets was an emotional resource. Technical aids were seen as a resource such as the walker that offered both indoor and outdoor security. Other equipment such as personal alarms or being easily heard by phone with both social- and professional networks also promoted the experience of security. The professional network was a resource, and this needed to be available when the patient needed / wanted help and support where the patient’s own abilities were not sufficient. It was perceived as a security to have access to assistants / enrolled nurses, registered nurses and the same physician who could make home visits. Getting out in a safe and secure way with the support of the home care service was also perceived as a security. Personnel who had the time and interest to listen were a further source of experienced safety.

### Structural analysis

Quotes from the interviews are presented with the informants’ ID in parentheses. The patient is then named with a number and relatives with an A before the number. Within the statements, we could discern two different main areas, personal resources, and homecare.

### Main area 1: the person/patient’s self-care resources

The area of ​​self-care resources could in turn be divided into themes - personal, social, and technical resources that contribute as success factors in self-care for the older persons with multiple diagnoses based on the interviewees’ narratives from everyday life in the homecare situation.

### Personal resources

The findings showed that the success factors for the possibility of staying at home despite having multiple care needs as described by both patients and their relatives, related to personal resources that the multi-diagnosed older person had even before becoming ill and care dependent. These personal identified resources had often been present as part of the person throughout their life. The personal resources were not dependent on external circumstances or the structure of professional efforts. In general, more patients mentioned resources of an impractical nature and emphasized emotional security as an important part of the personal resources that need to be available to facilitate homecare.

#### Belief in the future

was another part of the personal resources described. Keeping a positive view on life and what life would bring. “*No*,* you have to try to make the best of it. And then there is that*,* whether it is positive or negative*,* it must be so.*” (7). The belief in the future of the patient also became an incentive for relatives to hold on to the desire to make homecare work. Staying at home despite multiple care needs was done in consensus with the spouse/ cohabiting relatives. Even if, out-of-town children with a more peripheral contact might have more conflicting opinions against the patient’s strong desire to remain at home. “*As long as xx is doing reasonably well*,* I’m fine and feel hopeful that we’ll be fine at home*.” (A1) Both relatives ‘and patients’ **hopes** for stability or at least to avoid deterioration was also a success factor. Many testified to an optimistic view of their situations and occasionally dreamt of a situation where everything was for the better. Several patients felt more hopeful than the doctors they met expressed: “*We do not have much more to do (the doctor at VC). And then you think -shit*,* do not say it*,* there is a little more*,* right*?” (9).

#### Religiosity and spirituality

were factors of faith which gave strength in difficult situations for patients and relatives. Faith could reduce any anxiety with a confidence that everything was as it should be regardless of the circumstances. “*I’m not afraid of anything. If the Lord wants*,* he will take me home*,* then. Let’s see what happens* [laughs]. *That’s about how I feel*.” (5).

Patients mainly mentioned **love** as part of a person’s self-care resources. Feeling loved, receiving and giving love in relationships gave strength in continuing homecare. Love also gave a positive atmosphere even if libido failed to function in everyday life. “*We have never scolded each other*,* but… No*,* it’s just been loving words all along. This is something you have time to think about when you lie there and do not have the strength to get up once*,* you know. Damn*,* how lucky I really am. No*,* she’s worth her weight in gold. It’s just like that.”* (4).

Informants (both patients and interviewed relatives) also testified about the importance of continuing to keep their loving relationships alive, meaning they did not want to go to an institution such as a hospital. “*He is my best friend and husband and everything to me*,* so to speak*,* I have everything in him.”* (A1).

Love was an important driving force to stay at home despite multiple difficulties, love gave strength to fight for keeping the living together relationship both by patients and their relatives / partners.

### Social resources

A well-functioning social network in the form of friends and acquaintances was a success factor, even though the very oldest in the informant group began to feel that this very resource that had been their help and support earlier in life began to thin out. With old age, friends had died or disappeared in other ways. However, some testified about the possibility of making new ties even at an advanced age. “*I’ve found an old friend. It was quite fun; we have a lot in common. Calling each other and… I cannot get there… I cannot get anywhere more than they push me. But she can get here*,* that’s something very positive. Otherwise*,* you would be much more alone. We are old and weak*,* but we have good contact. That is probably what is positive. My old friends*,* otherwise*,* they’re gone.”* (7).

Having friends, human or animals could bring security and company and reduce loneliness. Having a dog was also described as something that spiced up life even for the cohabiting relative. Otherwise, having a multi-sick relative could feel a bit like being locked up. Having a dog gave space and physical exercise for the cohabiting person. Dogs were also able to normalize the situation to become not so diametrically different from life before the ill-health became apparent: “*I have everything I can think of. She does not want to sell*,* and I say*,* then we do not. She wants this left*,* and the dog and everything*,* whatever happens. So*,* when I lie down*,* then both (the dogs) lie down as well*.” (5).

### Technical resources

Various technical resources could contribute as success factors for the multi-sick people and their relatives. Partly as a pastime and company such as TV but also as security-creating factors. “*My company is the TV. It’s incredible. It can be on. You do not have to look at everything as luck would have it*,* but then you can choose the best programmes.*” (7) One of the most mentioned security-creating technical resources was the walker, which made it possible to get around both inside and outside the home with a reduced fear of falling. One of the patients put it this way: *“Yes*,* I have a friend… Yes*,* it’s my friend now*,* the walker. It’s out here and in here*,* I have two. So*,* I have an outdoor walker and indoor walker now. It is so good. It helps a lot that I can shop a bit too and everything like that. That I do not… Depend on someone else to shop out there. So that’s a big help*,* the walker [laughs] I did not want it*,* no. I’m grateful it’s there.”* (6).

Similar statements were mentioned concerning having a permobile making life with disabilities a bit more flexible.

Being able to use a personal alarm whenever needed and being able to reach someone was another technical tool that promoted feeling safer at home despite multiple difficulties: A relative illustrates through the following quote what makes you feel safe and secure in your situation at home both as a relative and as a patient in the form of alarms in combination with the close-care team: “*He knows I can call there if I think he’s not feeling well and that he has this security alarm.*” (A2).

The identified personal, social, and technical resources of self-care were underlined when the interviewees talked about what they understood as success factors of homecare.

### Main area 2: care

The area of care could be divided into the themes - informal and professional care that contribute as success factors for the older persons with multiple diagnoses in homecare.

### Informal care

The informal care the relatives offered was a strong factor for the patient to be able to remain in their home. Almost all informants described a situation where relatives were of great help in their everyday life, as described in the following quote: " *Here comes my son*,* the one that must wash for me. That’s it*,* so that… If X hadn’t lived over there and had been able to help me*,* then I wouldn’t have been able to stay here.”* (3).

But it was not only relatives who served as informal carers. Neighbours also constituted a security and offered informal care. Sometimes they could help with acutely difficult situations and solve them so that the patient did not have to contact healthcare or other professional services: “*Then a neighbour comes down here at… I said - I must get help to get home*,* because I can’t get the hell home. I am completely exhausted”. “Yes*,* but we’ll fix that*,*” she said. She is so friendly. So*,* she followed me home again.* (2)

The biggest informal care effort was carried out by the partners/spouse of the multi-ill elderly. Many informants testified to the enormous amount of work they performed and sometimes to how tired their partners were: “*She does a great job*,* you know. She works… Or yes*,* she works two nights a week. So that… How she copes… I can see from her that she is getting tired over the years. The wife is the best when it comes to having security*,* stability*,* and joy. Then comes the interprofessional team because they cheer everything up. They see me every day and what ups and downs it is.”* (5).

In several interviews, the opinion was expressed by the patients that the partner/spouse was the single greatest cause for being able to stay at home, their impact was immeasurable compared to other resources described. Several of the relatives, however, emphasized the importance of receiving the support of the close-care team to be able to stay and carry out the informal care which was so important for the multi-ill persons who participated in the present study.

### Professional care

The staff acted professionally according to the interviewees while meeting the patients’ most basic needs such as food and personal hygiene by caring for the whole person. “*The staff cook porridge and are responsible for giving medicine in the morning. I get help with showering every 14 days. Unable to shower by yourself. I don’t feel safe otherwise since I fell.”* (3) As a consequence of the multimorbidity one can prefer to wash oneself with washcloths or similar between the showers. Other basic needs in relation to personal care were mentioned such as getting help with the support stockings to maintain circulation in the legs. “*Yes*,* I guess I’m still a bit anxious*,* they lubricate every night*,* you know*,* and put on support socks.”* (3).

All interviewees with multiple diagnoses had extensive medication with a lot of different tablets to administer. Getting help with the medications was experienced as creating security for both the patient and their relatives, which is described in the following relative interview: “*For a while I was a bit worried when he had problems with tablets and it wasn’t easy…Yes*,* he himself probably lost a bit then so that he kept…fainted several times then because he was given the wrong medication. But after that*,* the close-care team took over the medication and took care of everything like that… so now everything like that works. So*,* it feels calmer.”* (A3).

One factor in the professional care that was described as important was the continuity that could be offered by the close-care team, not least receiving home visits from the same staff as highlighted in the following relative interview: “*I think it feels better for another as well then as if you look at this closeness… relatives’ aspect that we know that the fewer… the more he recognizes*,* the better it is for him too*,* he feels a trust in them too. Getting new people all the time*,* it doesn’t give that confidence*,* but then… Before I thought he got into this*,* it was probably… that brings him a little more trouble*,* almost… Yeah*,* it was this that gets this connection with people. Now he knows… He recognizes most of them when they come instead*,* so that… And they probably have a little more time today too.”* (A3).

Having access to a phone number that both relatives and patients knew would be answered by the team they already knew and that they know their situation was a precondition of feeling safe and secure at home despite multimorbidity. *“If there is something I’m worried about*,* there are always people you can call and get an answer straight away*,* have someone to talk to instead… Yes*,* I don’t know how to explain*,* it’s only positive*,* a big relief.”* (A5).

It was also known that the staff who responded would take time to remedy the situation. From relative interview 2: “I feel very safe. But just the thought that you don’t need that much to feel safe. Like I just know they’re there and I can call when… If I’m worried about something, they always come.” Precisely knowing that there are resources not only for continuous care but also for emergency supervision is comforting.

### Discussion and comprehensive understanding

Though various health care programmes have been identified for persons with multiple diagnosis [6, 7, 9], when patients and their relatives in this study talk about success factors for being able to stay at home, they mainly talk about factors not linked to professional care rather to the person’s self-care resources. Such personal resources (belief in the future, hope, love, and spirituality), social resources (neighbours, friends, and pets) and technological resources (TV, alarms and walkers) [See also, 10] were the self-care resources providing the main support system of everyday life.

Something you might not always focus on when talking about the needs of older persons with multiple diagnoses came out clearly in the present study, namely how love and close relations was a major reason for wanting to continue close care at home and the close relation was a spur not to give in to life’s health difficulties. The importance of continuing one’s loving relationship and not wanting to be institutionalized was an important driving force keeping up the work both for the patients and their relatives/partners. The love aspect as a cause of wanting homecare instead of residential care seemed to be far from the care providers’ focus, as also Fry et al. [25] clames, who might reduce the older person to their physical health situation where the person’s mental health, social health and the older person’s own values ​​were sometimes forgotten. Valuing love more than health aspects underlines the importance of a more person-oriented [17–19] view on older persons’ lives and needs.

The informal care provided by relatives was also a significantly more central part of the participant’s descriptions of experienced success factors than the professional care. Previous research [26] in the same way shows informal care as a safety factor through the provision of alert and actionable care by loved ones, including spatial safety, that cannot be revised by the professional care the municipality can offer. The professional care is simply not enough for an older person with multiple needs nor any older person even though it is according to the view of earlier research [17–19] considered person-centred. However, the fact that self-care resources are the main success factors in homecare, should not be understood as meaning that it is unnecessary to develop professional care. Rather, professionals should explore and put even more effort into supporting the patient’s own self-care system of personal, social, technical resources and investigate the informal care provided.

Professional care as a resource was of course also important for the participants. This was especially the case for those who did not have close relatives and could not use informal care to the same extent as those who, for example, lived together with close relatives. The interprofessional team was, however, also important for relatives, as also shown in earlier studies [14, 18]. Several relatives emphasized the importance of having the support of the interprofessional team, working in a person- centred way to be able to carry on with the informal care.

When the interprofessional team communicated that they had time and could visit the patient at any time, relatives’ confidence in supporting the person to continue homecare over institutional hospital care was enhanced. Ironically, by generously offering interprofessional team time, less time for professional care was asked for.

### Limitations

The fact that all informants came from the same municipality, with a special interprofessional team consisting of regional and municipal caregiver organisations, with a focus on older persons with multiple diagnoses, may restrict the transferability to other municipalities with a less developed organisation for this target group. Price and Murnan [27] considered this limitation in their study design when following up the work of an integrated interprofessional team, that was designing new ways of working. Gathering patients and their relatives in the same analysis can be questioned, but the analysis aimed at illustrating the meaning of what Ricoeur [22] calls the phenomena which is the success criteria for being able to stay at home, not the description of the single experienced life situation for older persons with multiple diagnoses. Restricting the number of informants also meant receiving more extensive material to answer the aim and meaning of the phenomena.

## Conclusions

The main reason for being able to continue homecare is the person’s so-called self-care system: the personal, social, and technical resources which made the life situation bearable. Development of professional care is to anchor the team work to the patient’s own system of self-care including the available informal care.

## Data Availability

The datasets generated and/or analysed during the current study are not publicly available due to limitations of ethical approval involving the patient data and anonymity but are available from the corresponding author on reasonable request.
